# Wire Bow In Situ Measurement for Monitoring the Evolution of Sawing Capability of Diamond Wire Saw during Slicing Sapphire

**DOI:** 10.3390/ma17092134

**Published:** 2024-05-02

**Authors:** Zixing Yang, Hui Huang, Xinjiang Liao, Zhiyuan Lai, Zhiteng Xu, Yanjun Zhao

**Affiliations:** 1 Institute of Manufacturing Engineering, Huaqiao University, Xiamen 361021, China; 22011080017@stu.hqu.edu.cn (Z.Y.); huangh@hqu.edu.cn (H.H.); 1611403003@stu.hqu.edu.cn (Z.L.); 20011080016@stu.hqu.edu.cn (Z.X.); 2 College of Mechanical Engineering and Automation, Huaqiao University, Xiamen 361021, China; 3 Collaborative Innovation Center of Advanced Manufacturing Technology and Equipment for Stone Industry, Xiamen 361021, China; 4 State Key Laboratory for High Performance Tools, Zhengzhou Research Institute for Abrasives & Grinding Co., Ltd., Zhengzhou 450001, China; zyj@zzsm.com

**Keywords:** wire bow, sawing capability, in situ measuring, wire wear, diamond wire saw

## Abstract

Electroplated diamond wire sawing is widely used as a processing method to cut hard and brittle difficult-to-machine materials. Currently, obtaining the sawing capability of diamond wire saw through the wire bow is still difficult. In this paper, a method for calculating the sawing capability of diamond wire saw in real-time based on the wire bow is proposed. The influence of the renewed length per round trip, crystal orientation of sapphire, wire speed, and feed rate on the wire sawing capability has been revealed via slicing experiments. The results indicate that renewing the diamond wire saw, and reducing the wire speed and feed rate can delay the reduction in sawing capability. Furthermore, controlling the value of renewed length per round trip can make the diamond wire saw enter a stable cutting state, in which the capability of the wire saw no longer decreases. The sawing capability of diamond wire saw cutting in the A-plane of the sapphire is smaller than that of the C-plane, and a suitable feed rate or wire speed within the range of sawing parameters studied in this study can avoid a rapid decrease in the sawing capability of the wire saw during the cutting process. The knowledge obtained in this study provides a theoretical basis for monitoring the performance of the wire saw, and guidance for the wire cutting process in semiconductor manufacturing. In the future, it may even be possible to provide real-time performance parameters of diamond wire saw for the digital twin model of wire sawing.

## 1. Introduction

With the rapid development of the electronic information industry, semiconductor materials have become the cornerstone of the photovoltaic and integrated circuit field. The semiconductor ingots will be processed into wafer substrates through slicing, grinding, and polishing in sequence. Slicing is the first process of the whole production process, which determines the processing efficiency of subsequent grinding processes. The electroplated diamond wire saw is most widely used as the cutting tool for slicing silicon [1,2,3,4,5,6,7,8,9,10], sapphire [11,12,13,14,15], and SiC [16] due to its narrow saw kerf, low environmental pollution, and high processing quality. During the slicing process, diamond wire saw inevitably undergoes wear, which further reduces the capability of wire sawing and the quality of the wafer. Therefore, it is important to evaluate the sawing capability of the wire saw during the slicing process.

In the actual sawing process, both the properties of the material and the cutting parameters, including the wire speed, feed rate, wire tension, and renewed length per round trip, will influence the wear rate of the wire saw, which in turn affects the sawing capability. In the early stage of the study, researchers attempted to unveil the pattern of changes in the capability of diamond wire saw during the slicing process just by observing the wire wear offline. Although this method can accurately obtain the laws of change in the abrasive grain [17,18,19,20,21,22] and the diameter size [23,24,25,26,27] of the wire saw via the camera, SEM, or wire diameter optical inspection instrument, it is only able to roughly estimate the degree of wire wear after machining. And it is not possible to quantitatively characterize the sawing capability during machining, especially describing the change in sawing capability due to wear.

Qiu et al. [28] analyzed the relationship between the wire sawing capability and the wire bow, material removal rate, and sawing power. Kim et al. [17] clearly defined a physical quantity to describe the wire sawing capability during the slicing process for the first time. In their work, the wire sawing capability was related to the vertical sawing force and the depth of cut, and calculating the wire sawing capability required to measure both the wire bow and the vertical sawing force. This method enabled the detection of the wire sawing capability throughout the entire processing, but it required the use of two types of sensors, which increased the cost of detection. Coustier et al. [29] defined the wire sawing capability by the ratio of the abrasion coefficient to the kerf width, and it could finally be calculated by measuring the wire bow by the eddy current sensors [28,29] just in the steady-state sawing process. It made real-time detection of wire sawing capability possible by only monitoring the wire bow during steady-state slicing processing. However, this method was not suitable for unsteady-state slicing processing. The slicing processing included both steady-state slicing processing and unsteady-state slicing processing. Therefore, it is currently not possible to measure the wire bow only during the sawing process in order to calculate the sawing capability of the wire saw for the whole process in the field of evolution of the sawing capability of diamond wire sawing.

Changes in the sawing capability of the wire saw are reflected in the wire bow. Researchers have also established various mathematical models for the wire bow. Liedke et al. [30] developed an analytical model of macro-mechanical conditions during wire sawing, which relates typical process parameters to the shape and sawing force of the wire bow. Lin et al. [31] used a high-speed video camera to photograph wire bow and developed a simulation model of wire bow that considered the wire bow deflection curve as a quadratic curve. Xu et al. [32] established a numerical simulation model in wire saw machining, and the model correlates the wire bow angle with the process parameters. Dong et al. [33] established an analytical model for wire bow analysis between the process parameters and the wire bow based on the asymmetric arc hypothesis of the wire bow model, in which, when the sawing capability of the wire saw decreases, the asymmetry of the wire bow is enhanced, and the cutting stability is weakened. Lai et al. [34] established a dynamic model of wire sawing, which can well replicate the wire saw process, in which the sawing capability of the wire saw, the process parameters, and the size of the workpiece are the input parameters of the model in its formulation.

The above studies have shown that the wire bow comes from the flexibility of the wire saw and that the value of the bow is influenced by the size of the workpiece being machined, the sawing capability of the wire saw and the variation in the process parameters. In the case of round workpieces, the arc length of the contact between the workpiece and the wire varies during the machining process; furthermore, the process parameters may change during sawing depending on the setting, and these can lead to frequent changes in the wire bow during the sawing process. Therefore, the value of the wire bow often reflects a nonstationary sawing process. Because of this, it is inaccurate to calculate the sawing capability of the wire saw by relying solely on the value of the wire bow and ignoring the rate of change of the wire bow in the actual production process.

In this paper, based on the measured real-time wire bows and considering the real-time changes in workpiece and process parameters, a model to characterize the sawing capability of wire saw online is derived, an evaluating indicator to characterize the sawing capability of wire saw is proposed, and the influence of different factors on the sawing capability of wire saw is analyzed.

## 2. Modeling of the Sawing Capability of Wire Saw

**Figure 1:**
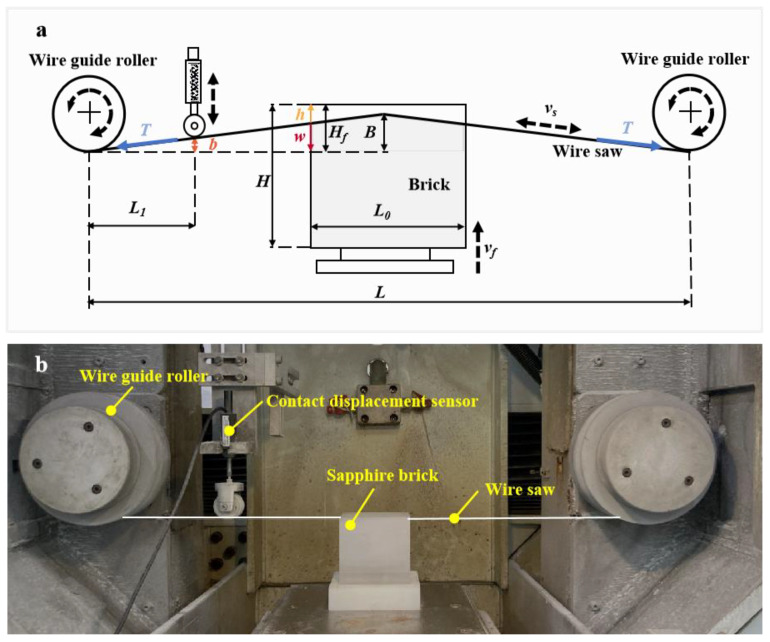
The diamond wire saw cutting device: (**a**) schematic diagram; (**b**) processing site.

The bending stiffness of the wire saw is neglected, and the wire saw is assumed to be a pre-stretched ideal string. The wire sawing system is simplified as shown in Figure 1a.

During the diamond wire sawing process, the wire saw moves at a speed of $v_{s}$, while the workpiece moves upward at a speed of $v_{f}$. The flexibility of the wire saw makes the existence of wire bow w. In Figure 1a, w is the size of the wire bow of the wire saw at the edge of the workpiece and B is the size of the wire bow of the wire saw in the middle of the workpiece. The workpiece is placed at the center of two guide rollers. Liedke et al. [30] have given the expressions as Equations (1) and (2) for the wire bow w during the machining process:

(1) $$\dot{w}+K\left(t\right)w=v_{f}(t)$$

(2) $$K\left(t\right)=\frac{4Tk_{p}v_{s}(t)}{L_{0}d_{w}(L-L_{0})}$$

where $v_{s}$: the absolute wire saw speed, $v_{f}$: the motion speed of the workpiece, $L_{0}$: the width of the workpiece, L: the spacing of the guide rollers, T: the tension on the wire saw, $k_{p}$: the Preston coefficient, which is inspired by the Preston equation [35], $d_{w}$: the diameter of the wire saw.

For this wire saw sawing process, when some of the parameters do not vary with time and the wire bow varies from 0, Liedke et al. [30] have given Equation (3) to describe the relationship of the wire bow *w* on the side of the workpiece with time:

(3) $$w\left(t\right)=\frac{L_{0}d_{w}\left(L-L_{0}\right)}{4Tk_{p}}\frac{v_{f}}{v_{s}}(1-e^{-\frac{4Tk_{p}v_{s}t}{L_{0}d_{w}\left(L-L_{0}\right)}})$$

From Equation (3), it can be seen that a change in any of the parameters in Equation (3), during the sawing process of the wire saw will result in a change in the bow of the wire perpendicular to the workpiece. Liedke et al. [30] showed that in Equation (3), the parameters related to the process parameters are $L_{0}$, $v_{s}$, $v_{f}$, L and T. When the wire saw is worn, the sawing capability of the wire saw changes and $k_{p}$ and $d_{w}$ will change; therefore, this paper defines the wire saw sawing capability as follows:

$$k_{f}=\frac{k_{p}}{d_{w}}.$$

And Equation (3) can be written as Equation (4):

(4) $$w\left(t\right)=\frac{L_{0}\left(L-L_{0}\right)}{4Tk_{f}}\frac{v_{f}}{v_{s}}(1-e^{-\frac{4Tk_{f}v_{s}t}{L_{0}\left(L-L_{0}\right)}})$$

From Equation (4), it can be seen that when the sawing capability of the wire saw increases, the wire bow decreases and the time to reach steady-state sawing decreases; when the sawing capability of the wire saw decreases, the wire bow increases and the time to reach steady-state sawing increases. From Equation (4) in the time domain, which cannot be directly separated from the sawing capability and the direct relationship between the wire bow, the Laplace transform is applied to Equation (4). The form of the Laplace transform of the output wire bow can be expressed as

(5) $$W_{O}\left(s\right)=\frac{W_{o}\left(s\right)}{W_{i}\left(s\right)}\cdot W_{i}\left(s\right)=\frac{L_{0}\left(L-L_{0}\right)}{4Tk_{f}}\frac{v_{f}}{v_{s}}\frac{1}{(\frac{L_{0}\left(L-L_{0}\right)}{4Tk_{f}}\frac{1}{v_{s}})s+1}$$

Stripping $k_{f}$ from the wire bow Equation (5) in the process of the Laplace transform.

Sign $A_{1}=\frac{L_{0}}{4T}\cdot \frac{v_{f}}{v_{s}}\cdot (L-L_{0})$, $A_{2}=\frac{L_{0}}{4T}\cdot \frac{1}{v_{s}}\cdot (L-L_{0})$

(6) $$W_{O}\left(s\right)=\frac{A_{1}\cdot \frac{1}{k_{f}}}{\left(\frac{1}{k_{f}}\cdot A_{2}\right)s+1}=\frac{A_{1}}{A_{2}\cdot s+k_{f}}$$

$k_{f}$ can be expressed as

(7) $$k_{f}=\frac{1-\frac{W_{O}\left(s\right)\cdot A_{2}s}{A_{1}}}{\frac{W_{O}(s)}{A_{1}}}$$

For $k_{f}$ inverse Laplace transform to the time domain, $k_{f}$ can be expressed as

(8) $$k_{f}=\frac{1-\frac{d(\frac{w_{o}\left(t\right)}{v_{f}})}{dt}}{\frac{w_{o}\left(t\right)}{A_{1}}}$$

This is the formula for the wire sawing capability $k_{f}$, where $w_{o}\left(t\right)$ is the curve of the wire bow over time.

The wire bow w and B in Figure 1 have the following geometric relationship

(9) $$\frac{B}{w}=\frac{L}{L-L_{0}}$$

According to Equation (9), $w_{o}\left(t\right)=w(t)=B\left(t\right)\cdot \frac{L-L_{0}}{L}$

(10) $$k_{f}=\frac{1-\frac{d(\frac{w(t)}{v_{f}})}{dt}}{\frac{w(t)}{A_{1}}}=\frac{1-\frac{d(\frac{B\left(t\right)\cdot \frac{L-L_{0}}{L}}{v_{f}})}{dt}}{\frac{B\left(t\right)}{\frac{L_{0}}{4T}\cdot \frac{v_{f}}{v_{s}}\cdot L}}$$

where, B(t): the real-time wire bow corresponding to the middle of the output workpiece. In particular, when the feed rate does not vary with time during machining and the wire bow is in a steady state and does not vary with time, the wire sawing capability at this time can be expressed as

(11) $$k_{f}=\frac{L_{0}}{4T}\cdot \frac{v_{f}}{v_{s}}\cdot \frac{1}{B}\cdot L$$

Equation (11) agrees with the formula for wire sawing capability in the steady state cutting process calculated by Coustier et al. [29] using a wire bow B, which initially verifies the correctness of the study. Equation (10) compared to Equation (11) can characterize the real-time wire saw sawing capability, including the unsteady sawing process. Equation (10) is established on the condition that all parameters within the formula are kept constant. When the reciprocating wire saw in the sawing process enters the stage of uniform motion and, in the sawing process, the wire sawing capability of the small change in a reciprocal cycle can be regarded as approximately unchanged, the formula can be used to calculate the cycle of the wire saw sawing capability.

## 3. Experiments of Wire Bow Measurement

### 3.1. Experimental Setup

The experiments in this study were carried out on a single-wire sawing machine (JXQ-1201, No.45, Research Institute of China Electronics Technology Group Corporation, Beijing, China), as shown in Figure 1b. The workpiece was the sapphire block and fed upwards. The diamond wire saw made periodic reciprocating motion between the guide rollers, during which the diamond wire saw experienced forward acceleration, constant speed, deceleration, reverse acceleration, constant speed, and deceleration in sequence. In this sawing machine, a new wire feed motion could be achieved by controlling the distance of forward and backward movement of the diamond wire saw. 

**Figure 2:**
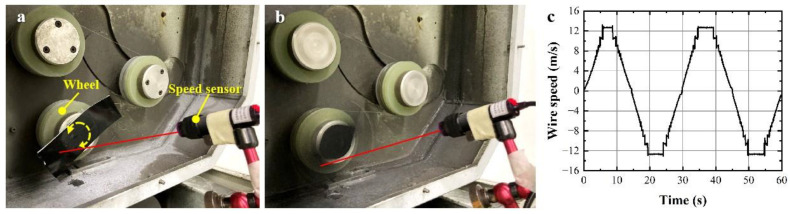
The schematic of the wire speed detection: (**a**) static and (**b**) dynamic; (**c**) the detected wire speed during sawing.

Before cutting experiments, the actual wire speed was measured by the photoelectric speed sensor, as shown in Figure 2a,b. Figure 2c gives the wire speed varying with time in two reciprocal cycles of the diamond wire saw in the fastest running mode, which indicates that the maximum permissible absolute wire speed of the machine is 13.3 m/s. By calculating, its corresponding absolute average wire speed in one reciprocal cycle is 6.67 m/s. The results of the preliminary experiments indicated that both excessive and insufficient wire speed could easily result in wire breakage, making the test end prematurely. The maximum feed rate allowed for cutting the sapphire block in this paper is 0.4 mm/min. Parameters related to the diamond wire saw cutting device are listed in Table 1.

Considering the practical circumstances, five series of experiments were ultimately designed to investigate the effects of the renewed length per round trip, crystal orientations of sapphire, feed rate, and average wire speed on wire sawing capability, as shown in Table 2. Experiments series 1 and series 2 were used to investigate the effects of the renewed length per round trip on the wire sawing capability. Experiments series 3, series 4, and series 5 were used to investigate the effects of the crystal orientation of sapphire, feed rate, and average wire speed on wire sawing capability, respectively.

### 3.2. In-Situ Measurement of Wire Bow and Processing of Wire Bow Data

As shown in Figure 1b, a small guide pulley was assembled under the spring of the contact spring-displacement sensor (GTS11A15, Taizhou Quantum Electronic Technology Co., Ltd., Taizhou, Zhejiang Province, China) to measure the value of the wire bow during the cutting process. The instrumental setup for the sensor is shown in Table 3. Before sawing experiments, the guide pulley was just in contact with the wire saw. The wire bow formed during the cutting process led to an upward displacement of the guide pulley, and the displacement was converted into a voltage value by the sensor. The output voltage of the spring-displacement sensor was collected by the data acquisition module (DH5981, Donghua Testing Technology Co., Ltd., Taizhou, Jiangsu Province, China) at a sampling frequency of 500 Hz. The sensor was calibrated before use to ensure that the output voltage matched the output displacement b.

**Figure 3:**
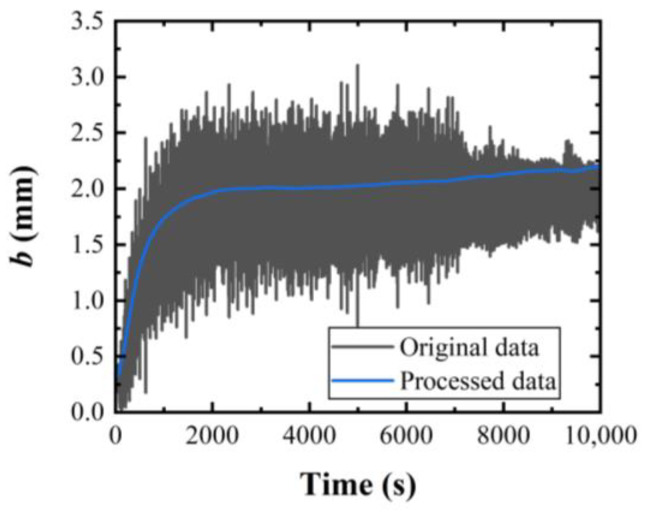
Comparison of original and processed data of the wire bow.

As shown in Figure 1a, through the geometric relationship, $\frac{w}{b}=\frac{L-L_{0}}{2L_{1}}$, the value measured by the wire bow sensor could be converted into the wire bow w. The original displacement data obtained by the wire bow sensor was first filtered using Fourier low-pass filtering, and then smoothed using the adjacency averaging method, to obtain the smooth curve of the wire bow. The wire bow data was taken at 4 s intervals. Figure 3 gives the original and smoothed signal curves of the wire bow. 

### 3.3. Calculation of Effective Sawing Depth

The change in sawing capability with time cannot fully reflect the relationship between sawing results and sawing capability during processing, so the effective sawing depth is used to reflect the sawing results, which in turn reflects the relationship between sawing results and the change in sawing capability.

As shown in Figure 1a, the effective sawing depth *h* can be calculated from the current feed displacement of the workpiece and the wire bow value, the height of the material removed by the wire saw is denoted as *h*, the total feed displacement of the workpiece is *H_f_*, and the wire bow value is *w*. The effective sawing depth can be expressed as

(12) $$h=H_{f}-w$$

## 4. Results and Discussion

### 4.1. Morphology of Diamond Wire Saw 

**Figure 4:**
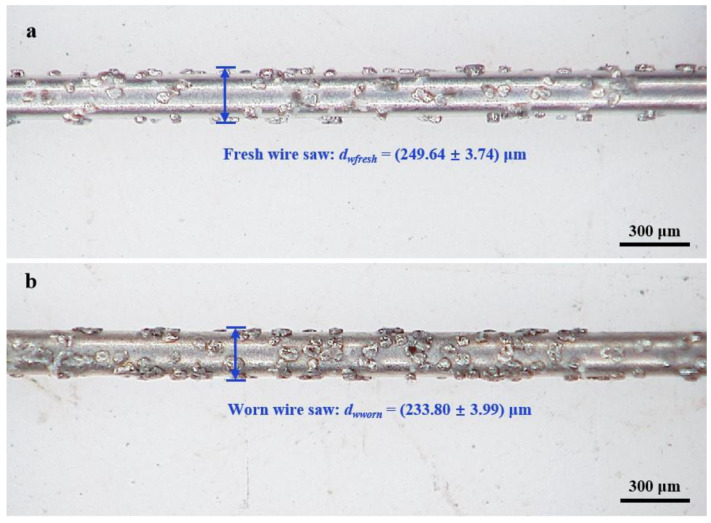
The morphology of diamond wire saw: (**a**) fresh and (**b**) worn.

The microstructure morphologies of fresh and worn diamond wire saws were observed using a 3D digital microscope (KH-8700, Hirox Co., Ltd., Tokyo Metropolitan, Tokyo, Japan), as illustrated in Figure 4. The average diameter of the new wire saw was approximately 250 μm, and the edge shape of the abrasive grains is clearly shown in Figure 4a; the average diameter of the worn wire saw was approximately 234 μm, and some of the abrasive grains were smoothed out as seen in Figure 4b. The wear of the diamond wire saw reflected the decrease in wire sawing capability.

### 4.2. Effect of Renewed Length per Round Trip on Wire Sawing Capability

**Figure 5:**
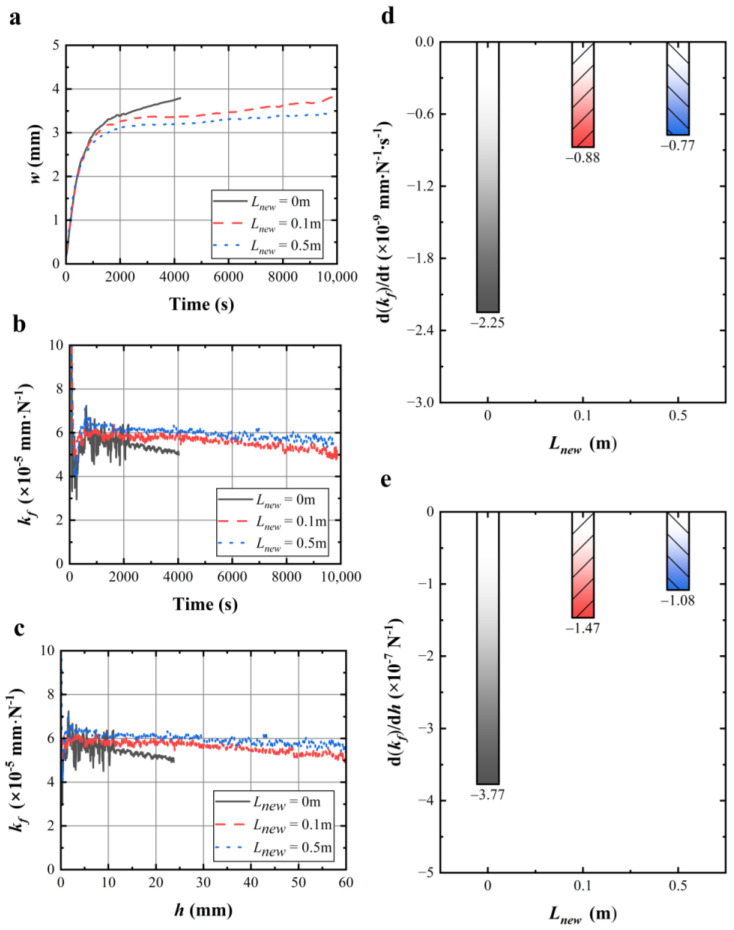
(**a**) Variation in wire bow with sawing time under different renewed lengths per round trip; (**b**) variation in calculated wire sawing capability with sawing time under different renewed lengths per round trip; (**c**) variation in calculated wire sawing capability with effective sawing depth under different renewed lengths per round trip; (**d**) histogram of calculated change rate of wire sawing capability from 2000 s to 10,000 s under different renewed lengths per round trip; (**e**) histogram of calculated change rate of wire sawing capability in the effective sawing depth from 10 mm to 60 mm under different renewed lengths per round trip.

Figure 5a shows the wire bow *w* varying with the renewed length per round trip, in which the wire bow *w* increases with the decrease in renewed length per round trip. Figure 5b shows the wire sawing capability over time, which was calculated from the wire bow curve in Figure 5a. In 0~10,000 s, the wire sawing capabilities were ranked as follows: *k_f_* (*L_new_* = 0.5 m) > *k_f_* (*L_new_* = 0.1 m) > *k_f_* (*L_new_* = 0 m). Figure 5c shows the wire sawing capability curve over the effective sawing depth. In the effective sawing depth of 0~60 mm, the wire sawing capabilities were ranked as follows: *k_f_* (*L_new_* = 0.5 m) > *k_f_* (*L_new_* = 0.1 m) > *k_f_* (*L_new_* = 0 m).

Figure 5d illustrates the change rate of the wire sawing capability in the sawing time of 2000~10,000 s at different renewed lengths per round trip, which is obtained from a linear fit to the data on wire saw capability. As shown in Figure 5d, all the change rates of wire sawing capability at different renewed lengths were negative, which indicates that the wire sawing capability declined with time. Furthermore, the decline rate of the wire sawing capability decreased with renewed lengths of diamond wire saw. Similarly, Figure 5e illustrates the change rate of the wire sawing capability with the effective sawing depth at different renewed lengths per round trip. In the effective sawing depth of 10~60 mm, the decline rate of the wire sawing capability decreased with the renewed length. It should be noted that the renewed lengths per round trip did not affect the wire sawing capability at the initial stage of the cutting process, as shown in Figure 5b,e. However, they affected the change rate of the wire sawing capability, which in turn affected the sawing capability of the wire saw during machining.

The experimental results obtained in Figure 5 are consistent with the findings of other scholars. Kim et al. [36] found that the cutting performance of wire saw varied with the renewed lengths per round trip. Yang et al. [37] found that the volume of material removal per unit length of the wire saw decreased and the wire saw wear decreased when the renewed length per round trip was increased. The experimental results clearly indicated that the wire sawing capability in the machining area can be improved by increasing the renewed length per round trip during the cutting process. 

**Figure 6:**
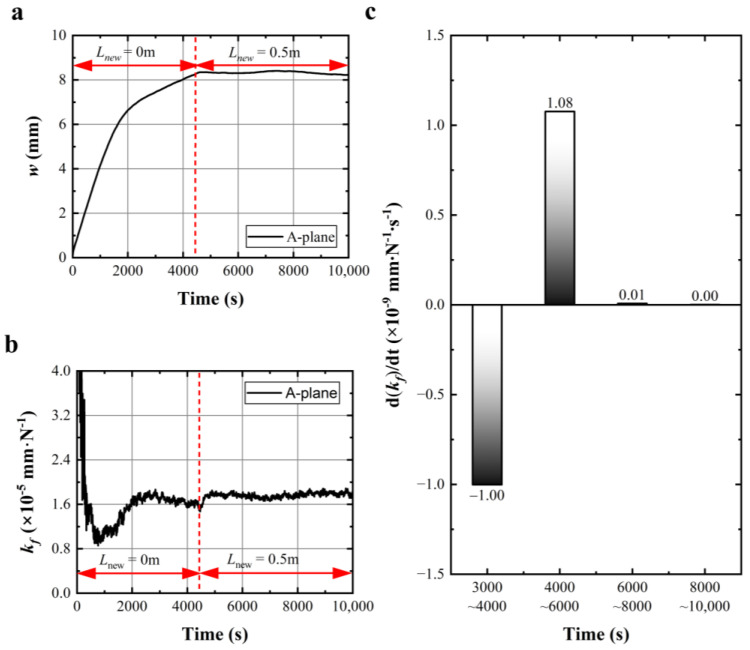
(**a**,**b**) Wire bow curve and calculated wire sawing capability with time under variable renewed length per round trip; (**c**) change rate of wire sawing capability in the relatively stable cutting stages.

Figure 6a shows the wire bow curve of sawing A-plane sapphire as the renewed length per round trip was adjusted from 0 m to 0.5 m during the sawing process. As the sawing process was without renewed lengths per round trip, the wire bow showed a linear increase trend in the initial sawing process and then increased slowly. As the renewed length per round trip was set from 0 to 0.5 m, the wire bow rapidly went to a stable state. During the whole sawing process in Figure 6a, the calculated wire sawing capability with time was given in Figure 6b. As shown in Figure 6b, the wire sawing capability rapidly decreased in the initial sawing process of 0~900 s; then it slowly climbed to a maximum value during 900~3000 s and showed a slow linear decline trend during 3000~4450 s; once the renewed length per round trip was set from 0 to 0.5 m, the wire sawing capability climbed and rapidly became stable. The wire sawing capability disturbance that occurred before 3000 s might be caused by an inevitable measurement error resulting from the spring displacement sensor.

Figure 6c shows the change rate of wire sawing capability in the relatively stable stages of the cutting process. During 3000~4450 s in the sawing stage without renewed length per round trip, the change rate of the wire sawing capability was negative, which indicated that the wire sawing capability declined with time resulting from wire wear during the cutting process. During 4500~10,000 s in the sawing stage with a renewed length per round trip of 0.5 m, the change rate of the wire sawing capability was near zero, which indicated that the increase in sawing capability brought by renewed lengths balanced with the decrease in sawing capability caused by wire wear, resulting in a stable cutting state. This set of experiments verifies the effectiveness of adjusting wire sawing capability by controlling the renewed length per round trip during sawing. 

### 4.3. Effect of Sapphire Crystal Orientation on Wire Sawing Capability

**Figure 7:**
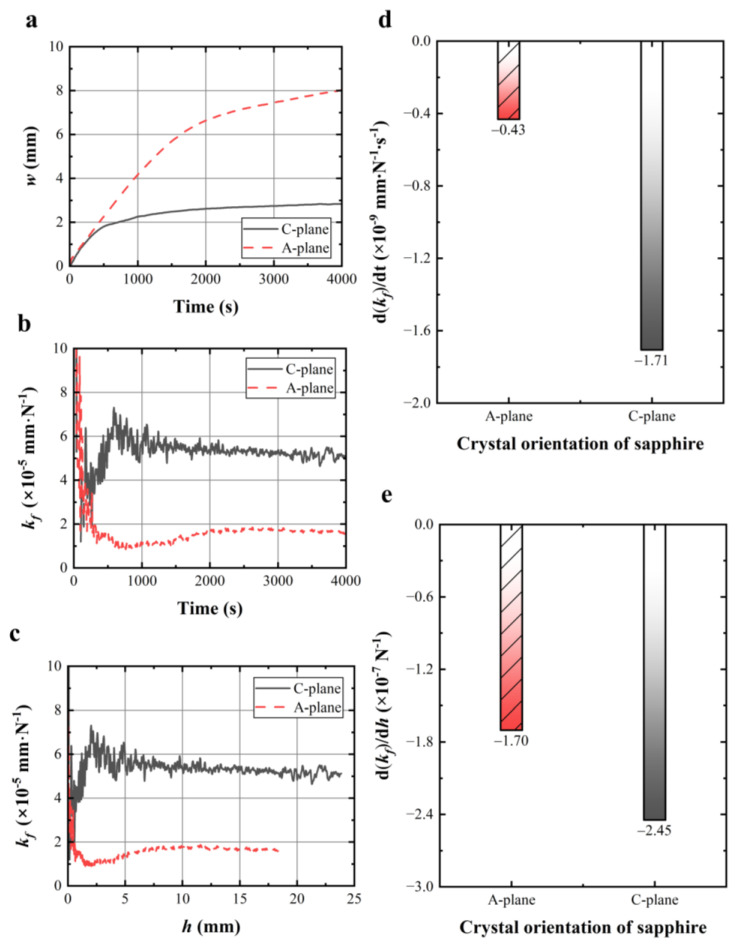
Under different crystal orientations of sapphire: (**a**,**b**) wire bow and calculated wire sawing capability with time; (**c**) calculated wire sawing capability with effective sawing depth; (**d**) histogram of the change rate of wire sawing capability from the 2000 s to 4000 s; (**e**) histogram of the calculated change rate of wire sawing capability from 10 mm to 25 mm.

Figure 7a gives the wire bow of sawing A-plane and C-plane sapphire under the same cutting parameters, in which the wire bow of sawing A-plane of sapphire is significantly bigger than that of sawing C-plane of sapphire. The wire sawing capabilities with the sawing time or the effective sawing depth in these two crystal facets were ranked as follows: *k_f_* (A-plane) < *k_f_* (C-plane), as shown in Figure 7b,c. The results above indicated that it was more difficult to saw the A-plane of sapphire than the C-plane of sapphire. This experimental phenomenon is consistent with the previous experimental results reported by Huang et al. [38], in which the tangential force on the sawing A-plane of sapphire was greater than that on the sawing C-plane of sapphire. The reason was attributed to the difference in the fracture strength of sapphire crystal A-plane and C-plane [13,39,40]. The fracture strength of the A-plane of sapphire is bigger than that of the C-plane, which indicates more energy consumption and a bigger sawing force and wire bow for the wire saw to achieve material removal in the A-plane. Figure 7d,e gives the change rate of the wire sawing capabilities of these two crystal facets with the sawing time and the effective sawing depth, respectively. The decline rate of the wire sawing capability value of the C-plane is more negative than that of the A-plane. 

### 4.4. Effect of Feed Rates on Wire Sawing Capability

**Figure 8:**
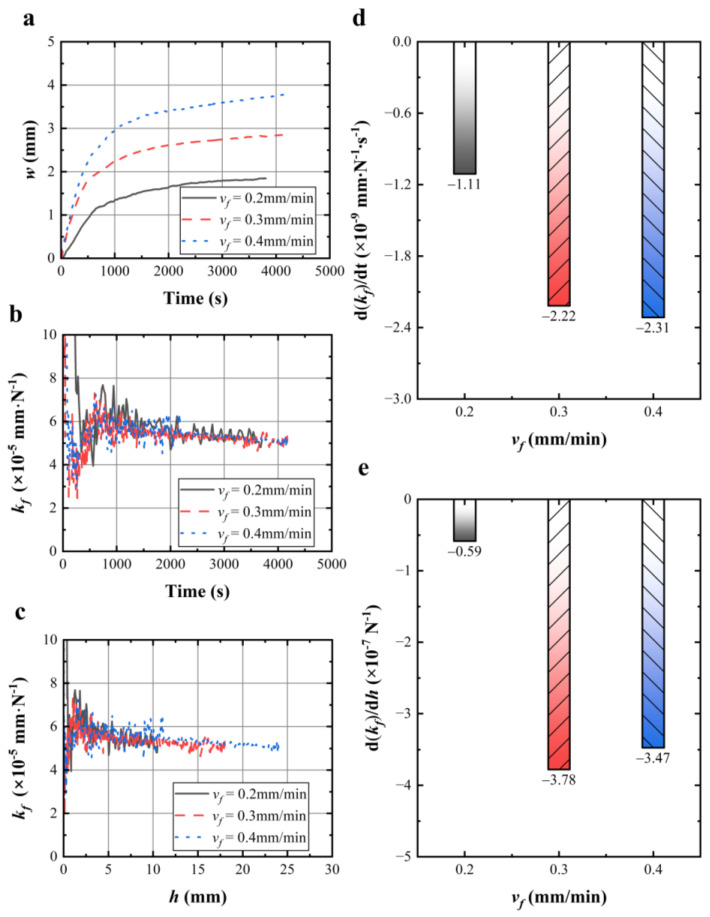
(**a**,**b**) Wire bow and calculated wire sawing capability with sawing time under different feed rates; (**c**) calculated wire sawing capability with effective sawing depth under different feed rates; (**d**) change rate of the wire sawing capability from 2000 s to 3700 s under different feed rates; (**e**) calculated change rate of the wire sawing capability from 5 mm to 20 mm under different feed rates.

The wire bow shown in Figure 8a increased with the feed rate during the cutting process. Figure 8b,c shows the effect of feed rates on the wire sawing capability with time and the effective sawing depth, respectively. Figure 8d,e illustrates the corresponding change rate of the wire sawing capability at different feed rates. As shown in Figure 8d, the change rates of the wire sawing capability were ranked as follows: (d(*k_f_*)/dt) (*v_f_* = 0.2 mm/min) > (d(*k_f_*)/dt) (*v_f_* = 0.3 mm/min) > (d(*k_f_*)/dt) (*v_f_* = 0.4 mm/min). The experimental results obtained in Figure 8d are consistent with the results reported by Kim et al. [17] that the wire sawing capability of the experimental group with a small feed rate decreases at a slow rate. The faster rate of decline in wire sawing capability increases with feed rate. As shown in Figure 8e, the change rate of wire sawing capability from 5 mm to 20 mm at different feed rates were ranked as follows: (d(*k_f_*)/dh) (*v_f_* = 0.2 mm/min) < (d(*k_f_*)/dh) (*v_f_* = 0.3 mm/min) < (d(*k_f_*)/dh) (*v_f_* = 0.4 mm/min). Different feed rates mainly affect the change rate of the wire sawing capability. Therefore, selecting the smaller feed rate will help to reduce the decline in wire sawing capability when sawing workpieces.

### 4.5. Effect of Average Absolute Wire Speeds on Wire Sawing Capability

**Figure 9:**
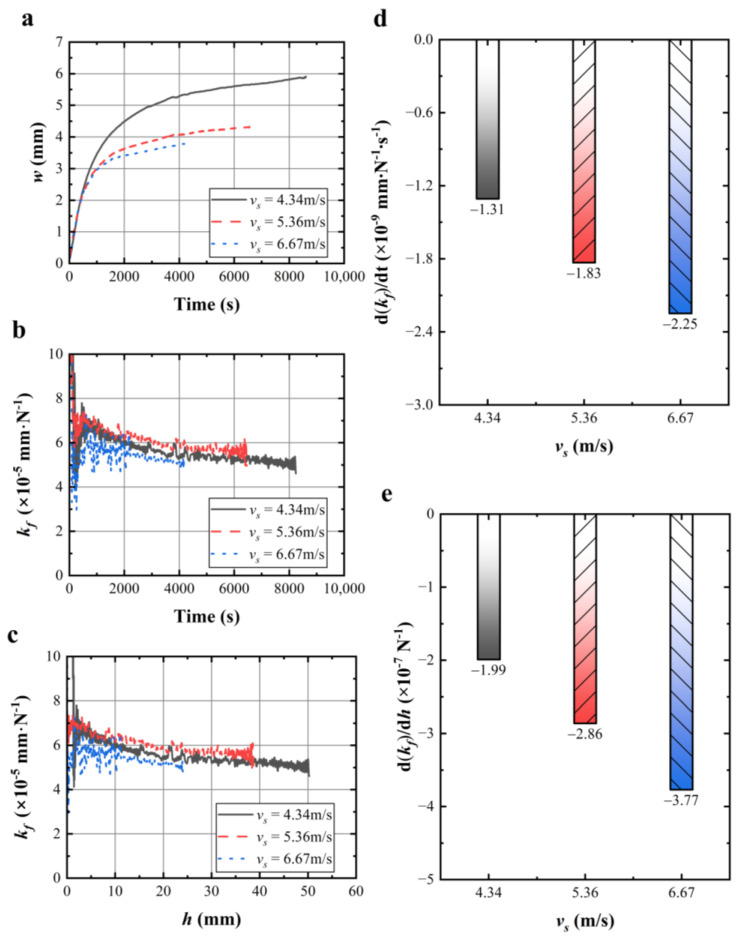
(**a**,**b**) Wire bow and calculated wire sawing capability with sawing time under different average absolute wire speeds; (**c**) calculated wire sawing capability with effective sawing depth under different average absolute wire speeds; (**d**,**e**) change rates of the wire sawing capability from the 2000 s to 8000 s and from 10 mm to 50 mm under different wire speed.

The wire bow shown in Figure 9a decreased with the increase in average absolute wire speed. As shown in Figure 9b, the wire sawing capability during 2000~8000 s was ranked as follows: *k_f_* (*v_s_* = 5.36 m/s) > *k_f_* (*v_s_* = 4.34 m/s) > *k_f_* (*v_s_* = 6.67 m/s). Figure 9c shows the wire sawing capability over effective sawing depth. From 10 mm to 20 mm, the wire sawing capabilities were ranked as follows: *k_f_* (*v_s_* = 5.36 m/s) > *k_f_* (*v_s_* = 4.34 m/s) > *k_f_* (*v_s_* = 6.67 m/s). Figure 9d,e illustrates the change rate of the wire sawing capability with time and effective sawing depth at different average absolute wire speeds. The decline rate of the wire sawing capability was ranked as follows: (d(*k_f_*)/dt) (*v_s_* = 4.34 m/s) < (d(*k_f_*)/dt) (*v_s_* = 5.36 m/s) < (d(*k_f_*)/dt) (*v_s_* = 6.67 m/s), which indicated the decline rate of wire sawing capability increased with the increase in average absolute wire speed within the small range of average absolute wire speeds studied in this study. The greater the average absolute wire speed chosen, the faster the wire saw capability decreased. Therefore, selecting a smaller average absolute wire speed can reduce the decline in the sawing capability of the wire saw.

## 5. Conclusions

This paper deduced a model of wire sawing capability and carried out sawing sapphire experiments to research the effect of sawing parameters on wire sawing capability. The obtained results in this paper can guide monitoring and regulating the sawing performance of the wire saw and might also provide a real-time parameter of the wire sawing capability for the digital twin model of the wire sawing process in the future. The main contributions of this paper can be summarized as follows:

1. A theoretical model of the real-time sawing capability of wire saw was developed, which could effectively characterize the effect of wire saw wear on machining online.
2. Under the same machining parameters, the wire bow decreased with the increase in the wire sawing capability. The wire sawing capability increased with the increase in the renewed length per round trip and wire speeds. The decline rate of the wire sawing capability decreased with the increase in renewed length per round trip, with the decrease in feed rate and wire speed.
3. The A-plane of sapphire was more difficult to cut than the C-plane of sapphire due to the higher fracture strength. The diamond wire saw exhibited a lower sawing capability when cutting the A-plane of sapphire.

## Figures and Tables

**Table 1:** Parameters related to the diamond wire saw cutting device.

Parameter		Short Name	Numerical Value
Wire motion	Variation range of the wire tension	*T*	35 N
	Average absolute wire speed	*v_s_*	0~6.67 m/s
	Wire forward length	*L_fw_*	100 m
	Wire backward length	*L_bw_*	99.5~100 m
	Renewed length per round trip	*L_new_ = L_fw_* − *L_bw_*	0~0.5 m
Wire guides	Guide rollers distance	*L*	486 mm
	Radius	*R*	115 mm
	Pitch of the grooves	*P*	1 mm
Sapphire brick (C-plane, A-plane)	Width	*L*_0_	65 mm
	Height	*H*	60 mm
	Feed rate	*v_f_*	0~0.4 mm/min
Wire saw	Diameter	*d_w_*	250 μm

**Table 2:** Wire sawing parameters for five series cutting experiments.

Series	Average Absolute Wire Speed *v_s_* (m/s)	Feed Rate *v_f_* (mm/min)	Renewed Length per Round Trip *L_new_* (m)	Sapphire Crystal
1	6.67	0.4	0, 0.1, 0.5	C-plane
2	6.67	0.4	0, 0.5	A-plane
3	6.67	0.4	0	C-plane, A-plane
4	6.67	0.2, 0.3, 0.4	0	C-plane
5	4.34, 5.36, 6.67	0.4	0	C-plane

**Table 3:** Instrumental setup for the contact wire bow sensor.

Sensor Indicators	Value
Type	Contact spring-displacement sensor
Measuring range	15 mm
Spring force	1.9 N
Distance between sensor and guide roller *L*_1_	110 mm

## Data Availability

Data are contained within the article.
